# Serological Response to SARS-CoV-2 in Health Care Workers Employed in a Large Tertiary Hospital in Lombardy, Northern Italy

**DOI:** 10.3390/microorganisms9030488

**Published:** 2021-02-25

**Authors:** Agnese Comelli, Emanuele Focà, Emanuele Sansone, Cesare Tomasi, Elisa Albini, Eugenia Quiros-Roldan, Lina Rachele Tomasoni, Emma Sala, Carlo Bonfanti, Francesca Caccuri, Arnaldo Caruso, Giuseppe De Palma, Francesco Castelli

**Affiliations:** 1 University Department of Infectious and Tropical Diseases, University of Brescia and ASST Spedali Civili, 25123 Brescia, Italy; emanuele.foca@unibs.it (E.F.); eugeniaquiros@yahoo.it (E.Q.-R.); linatomasoni@yahoo.it (L.R.T.); francesco.castelli@unibs.it (F.C.); 2 Postgraduate School of Occupational Health, DSMC, University of Brescia, 25123 Brescia, Italy; e.sansone@unibs.it; 3 DSMC, University of Brescia, 25123 Brescia, Italy; cesare.tomasi@live.com; 4 Unit of Occupational Health, Hygiene, Toxicology and Occupational Prevention, ASST Spedali Civili, 25123 Brescia, Italy; elisa.albini@asst-spedalicivili.it (E.A.); emmasala08@gmail.com (E.S.); 5 Department of Microbiology and Virology, Spedali Civili, Piazzale Spedali Civili 1, 25123 Brescia, Italy; carlo.bonfanti@unibs.it (C.B.); francesca.caccuri@unibs.it (F.C.); arnaldo.caruso@unibs.it (A.C.); 6 University Department of Occupational Health and Industrial Hygiene, DSMC, University of Brescia and ASST Spedali Civili, 25123 Brescia, Italy; giuseppe.depalma@unibs.it

**Keywords:** SARS-CoV-2 infection, health care workers, COVID-19, seroprevalence

## Abstract

Background: COVID-19 pandemic is requesting unprecedented efforts by health-care workers (HCWs) in all countries, and especially in Italy during the first semester of 2020. Methods: This is a retrospective, observational study conducted at the Spedali Civili General Hospital, in Brescia, Northern Italy during the SARS CoV-2 pandemic in the first semester of 2020. Serum samples from HCWs were tested for SARS-CoV-2 spike protein-specific antibodies. An online survey was used to collect demographic, clinical, and epidemiological data. Results: Of the 1893 HCWs included, 433 (22.9%) were found seropositive for SARS-CoV-2 IgG. The cumulative prevalence of SARS-CoV-2 infection (antibodies production or past positive RT-PCR on nasal/throat swab) was 25.1% (475/1893). Fifty-six out of 433 (13%) seropositive participants declared to have been asymptomatic during the study period. The development of COVID-19 signs or symptoms is the main determinant of seropositivity (OR: 11.3, *p* < 0.0001) along with their duration and severity. 40/290 (14.5%) HCWs with documented positive RT-PCR during the study period did not show any detectable antibody response. IgG levels positively correlate with age, COVID-19-compatible signs and symptoms experienced and their duration. Conclusions: In this study, carried out in one of the most affected areas in Europe, we demonstrate that most HCWs with COVID-19 related symptoms develop a spike protein-specific antibodies with potential neutralizing effect.

## 1. Introduction

A novel Severe Acute Respiratory Syndrome Coronavirus 2 (SARS-CoV-2) was first detected in Wuhan (Hubei Province, China) in December 2019 following the appearance of a cluster of severe pneumonia cases that shared similar characteristics with SARS [1].

Given the rapid spread of cases in other countries throughout the world, the World Health Organization (WHO) declared the new coronavirus disease (COVID-19) as a pandemic in early March 2020.

Involving all types of health care personnel in the fight against the infection spread, SARS-CoV-2 pandemic is requesting unprecedented efforts by health care system in all countries around the world.

The incubation period may be as long as 2 weeks, most cases occurring 4–5 days after exposure to a COVID-19 case [2].

The rate of asymptomatic infections widely varies across studies: 40–80% [3,4] but transmission to close contact remains possible even if in the asymptomatic phase [5]. Early detection of asymptomatic infection as well as PPEs (personal protective equipment) availability and their correct use are crucial to control in-hospital outbreaks.

Most people infected with SARS-COV-2 develop specific antibody response approximately 1–2 weeks after the illness onset. As for most viral infection the seroconversion time of IgM and IgG antibodies appeared consequently. If IgM detection can provide a useful diagnostic tool, IgG identification and quantification could also be used to understand the epidemiology of SARS-CoV-2 infection and to define the level of immunity in patients [6,7].

Since late February to mid-April, Italy was one of the most affected western countries, accounting for more than 30,000 HCWs infected in 6 months of pandemic [8]. Likewise, Brescia (Lombardy region), accounting for more the 9000 COVID-19 cases and more than 2300 hospital admission on the 4 April 2020 [9,10], was one of the most affected provinces by SARS-CoV-2 spread.

Worldwide, health care workers (HCWs) are employed on the frontline to guarantee patients care and therefore exposed to a higher risk of acquiring SARS-CoV-2 infection in comparison to the general population [11]. On the other hand, if infected, they pose a risk for their colleagues and for vulnerable subjects (e.g., patients hospitalized for other reasons).

While the real effectiveness of different personal protective equipment (PPE) has not been established and the international recommendation still remains controversial [12,13], the SARS-CoV-2 spread among HCW, resulting in personnel shortage, would dramatically affect our capacity to tackle this pandemic.

Studies assessing seroprevalence in a specific community can provide important information on the spread of infection and can identify subgroups at greater risk than others.

In the context of a pandemic, such studies provide accurate quantification of the level of exposure among health care facilities workers and allow to identify categories or departments at higher risk.

Allocating adequate resources (i.e., personnel, PPEs and asymptomatic infections screening plans) to those department exposed to a higher risk of infection, is fundamental to respond with preparedness to possible recrudescence of the pandemic [14].

Several studies conducted in Europe, USA, and Asia report a wide range of SARS-CoV-2 antibody seroprevalence among HCWs with percentage from 0 to more than 30% with a significant rate of asymptomatic infection [11,15,16,17,18,19,20,21,22]. These results are difficult to compare because of the different laboratory methods employed (e.g., tests based on the recombinant nucleocapsid protein or directed to the receptor-binding domain of the spike protein) and the different epidemiological setting of each geographical area.

One of the main factors associated to SARS-CoV-2 infection in the hospital setting is PPE shortage or incorrected use [18,19]. A higher antibodies production has been detected among symptomatic subjects [11,15,19,22] as well as those workers employed in COVID-19 dedicated wards [11,20].

The primary objective of the present study is to evaluate, among HCWs of the Spedali Civili General Hospital in Brescia, Italy, the correlation between epidemiological and clinical characteristics reported by HCWs and positive serology for SARS-COV-2 (qualitative and quantitative results).

As secondary objective, we aim to investigate the rate of asymptomatic infections and the serological response against SARS-CoV-2 among the included HCWs.

## 2. Materials and Methods

### 2.1. Study Design, Population, and Design

This is a retrospective, observational study conducted at the Spedali Civili General Hospital, in Brescia, Northern Italy during the SARS CoV-2 pandemic in the first semester of 2020.

The Spedali Civili General Hospital in Brescia is a tertiary referral University teaching hospital with over 1500 beds and it supplies basic and specialized health services.

The study population included HCWs employed at the Spedali Civili General Hospital during the pandemic and it consists of those who deliver care or services to the patients: either directly as physicians or nurses, or indirectly as assistants, technicians, administrative officers, cleaning, maintenance, pharmacists, etc.

During the last week of April 2020 and May 2020 blood samples were collected for all the HCWs employed at the Spedali Civili General Hospital to assess the seroprevalence of SARS CoV-2 IgG antibodies.

The Human Resources Department provided to us all the available registered email addresses of HCWs to send them an online survey.

Then, they were proposed to fill in an online survey, investigating the following issues: demographics (age, sex), medical and pharmacological history, occupational information (job, working area, and hospital department), history of COVID-19 compatible signs or symptoms during the previous months, clinical evolution (hospitalization in ICU or in non-intensive ward, oxygen supply, radiologic evaluation, pharmacological treatment), date of onset and resolution of symptoms (only signs or symptoms that appeared from the 1 February 2020 until two weeks before serology testing), previous SARS CoV-2 RT-PCR testing by throat/nasal swab and history of close contact with suspected or proven COVID-19 cases (inside or outside the hospital).

The survey was available online from the beginning of June 2020 to the 31 July 2020.

Definition of close contact was defined according to WHO [23].

We define COVID-19 cases as mild if managed at home, as moderate if they needed hospitalization and severe if ICU admission was warranted.

Participants accessed the survey through a link sent to their e-mail and they completed the survey using a smartphone or computer.

Inclusion criteria were: being registered at the Spedali Civili Human Resources Department, being older than 17 years, and having answered to the online survey.

We excluded subjects who did not work during the pandemic or who did not give blood sample for serology testing because of whatever reason (e.g., sick leave, vacation, etc.).

In the analysis we considered as immunosuppressed every patient who reported to take any chronic steroids or another immunosuppressive treatment, participants with solid or hematologic cancer or suffering from autoimmune diseases.

### 2.2. Laboratory Procedures

During May 2020 all the HCWs gave blood samples to test SARS-CoV-2 antibodies.

Our hospital used the LIAISON^®^ SARS-CoV-2 S1/S2 IgG chemiluminescence serological assay, which can detect specific antibodies binding SARS-CoV-2 spike protein and the corresponding titer for each sample. Those antibodies demonstrated a positive correlation with neutralizing antibodies production [24].

The cut off of the employed assay were the following: positive if IgG level >15 AU/mL, uncertain if IgG level 12–15 AU/mL, negative if IgG level <12 AU/mL.

According to the manufacturer, the assay’s diagnostic sensitivity was 91.3% and 95.7% at >5 or ≥15 days from diagnosis, respectively. The assay’s specificity ranged between 97% and 98.5% [24].

Participants with serology uncertain results were included among seropositive subjects.

We collected retrospectively the serology results with IgG titer for each participant and the results of the SARS-CoV-2 RT-PCR on throat/nasal swab performed during the previous months (only symptomatic subjects or close contact with proven cased were tested during the study period).

### 2.3. Statistical Analysis

Statistical analysis was performed using the statistical software package IBM SPSS^®^ Statistics V25.0. We performed a descriptive analysis of the collected data. Categorical variables were described as frequencies and quantitative variables were expressed as mean, SD and range.

Variables’ relationships were investigated by the Chi-square or Fisher’s exact test (for categorical variables) and Wilcoxon rank test (for continuous variables).

Univariate analysis was employed to evaluate factors associated with the presence of antibodies against SARS-CoV-2.

Correlation analyses were performed by the Spearman’s correlation.

In this study we followed the strengthening the Reporting of observational studies in epidemiology (STROBE) guidelines.

## 3. Results

### 3.1. Baseline Characteristics

The Human Resources database includes 8093 HCWs. Email address was available for 3909 HCWs.

A total of 1893 subjects fulfilled the inclusion criteria, none of them were excluded.

The questionnaire participation rate was 48.2%.

The mean age was 44 years (STD 10.7) and 77.1% were female.

The three most represented professional categories were nurses (43.2%), physicians (21.7%), and support staff (14.3%).

About a half of the participants (1008/1893, 53.2%) worked in units admitting COVID-19 patients (general medicine or intensive care units—ICU) or at the emergency room (ER) during the study period.

Thirty-two percent of subject declared at least one comorbidity and 2.4% suffers from underlying immunosuppression.

**Figure 1:**
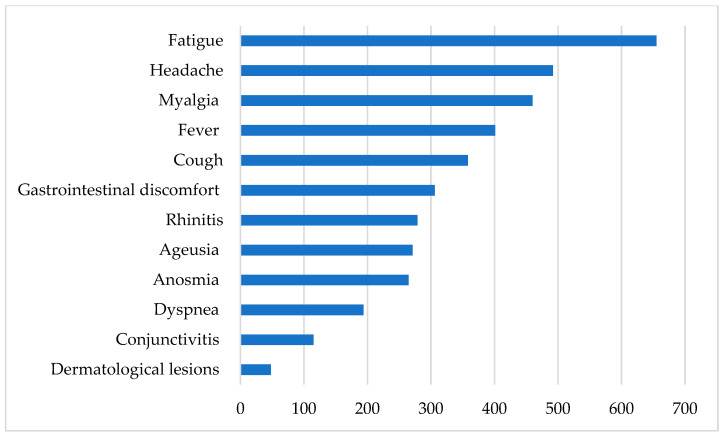
COVID-19 compatible signs and symptoms (*n* = 922).

Around half of HCWs recruited (922, 48.7%) reported having had COVID-19 compatible signs or symptoms during the study period. Fatigue (71%), headache (53.4%), myalgia (49.9%), and fever (43.5%) were the most common reported signs/symptoms (Figure 1).

The median duration of symptoms was 15 days (IQR 7–29).

One thousand two hundred ninety-four HCWs out of the study population had a RT-PCR of SARS-CoV-2 performed on nasal throat swab during the study period and 290 (22.4%) resulted positive.

Among symptomatic subjects, 4 were defined as severe cases (4/922, 0.43%), 20 as moderate (20/922, 2.2%), and the remaining patients suffered from mild symptoms and were managed at home.

Radiologic-confirmed pneumonia was reported in 48 cases out of 112 X-ray/CT scan (42.9%) performed because of severe symptoms.

Fifty-eight percent of recruited HCWs reported close contact with confirmed COVID-19 cases. For further data see Table 1.

### 3.2. Seroprevalence of SARS-CoV-2 S1/S2 IgG Antibodies

Four hundred thirty-three participants (22.9%) were found seropositive for IgG against SARS-CoV-2 S1/S2.

The Human Resources Department provide to us the seroprevalence among the 3909 HCWs whose email address was available: 17.3% (676/3909).

**Figure 2:**
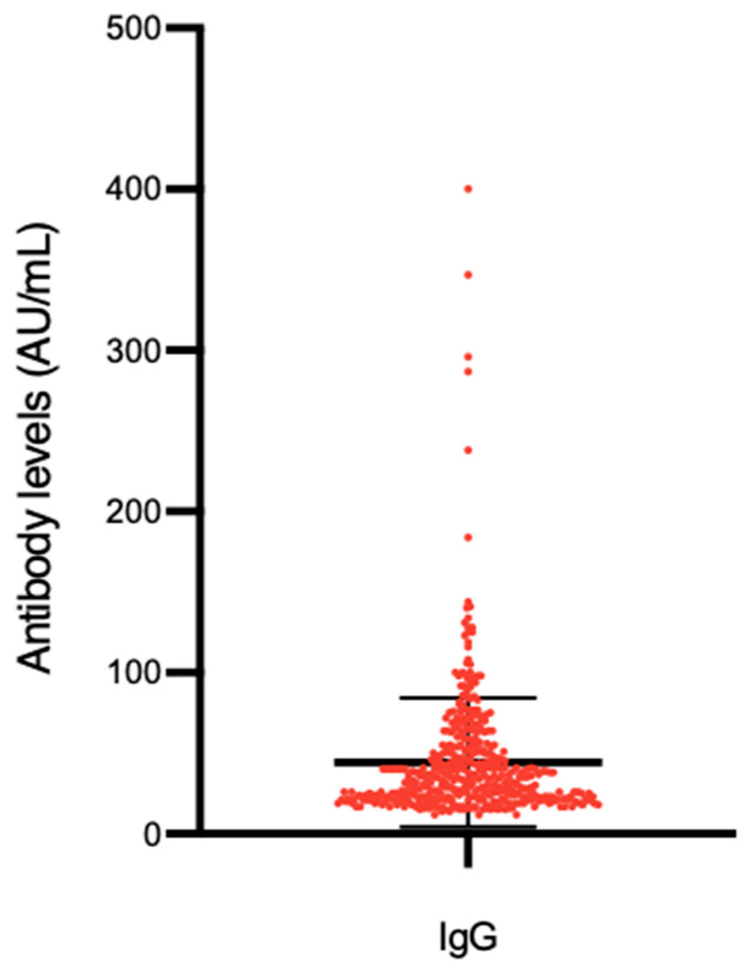
SARS-CoV-2 antibodies levels in seropositive participants (*n* = 433).

Distribution of antibody titers are presented in Figure 2.

Two hundred and ninety HCWs had been previously diagnosed with SARS-CoV-2 infection by RT-PCR in the study period and 42 of them (14.5%) did not show a detectable antibody response.

Three hundred and seventy-seven out of all seropositive participants (87%) reported having had COVID-19 compatible signs or symptoms during the study period, which results in an asymptomatic rate infection of 13% among seropositive participants.

The asymptomatic rate slightly decreases when we include the 42 patients with RT-PCR positive/serology negative among the infected population (56/475, 11.8%).

When considering as symptomatic only HCWs who suffer from the most suggestive and distinct symptoms and signs (fever, ageusia, anosmia, dyspnea, myalgia and cough), the rate of asymptomatic HCWs among the seropositive ones increase from 13% to 19.6%.

On the other hand, 545/1460 (37.3%) seronegative HCWs reported COVID-19 compatible signs/symptoms during the study period.

Participants who had had any COVID-19-compatible sign or symptom in the study period showed a higher likelihood of being seropositive (OR: 11.3, 95% CI 8.4–15.2, *p* < 0.0001) (Table 2).

Radiologic confirmed pneumonia (OR: 37.7, 95% CI: 9–156.4) and the need of oxygen supply (OR: 25, 95% CI: 3.4–193) positively correlated with positive SARS-CoV-2 IgG among symptomatic patients (both with a *p* < 0.0001).

Age, duration of signs and/or symptoms, positive SARS-CoV-2 RT-PCR on throat/nasal swab positively correlate with SARS-CoV-2 IgG production (Table 2).

HCWs who declared having had close contact with confirmed or suspected cases showed a statistically significant higher probability to result seropositive (OR: 1.5, 95% CI: 1.2; 1.9 and OR: 1.2, 95% CI: 1.7; 2.7, respectively) (Table 2).

Hospital admission because of moderate or severe COVID-19 showed a positive correlation with IgG production. Particularly, the strongest association with a positive title of IgG, was observed among those workers who required ward or ICU admission (both with statistical significance *p* < 0.0001).

Working in COVID-19 units/ER, professional category, age, sex, comorbidities, or any underlying immunodepression did not show any statistically significant association with presence of SARS-CoV-2 antibodies (Table 2).

The clinical pictures more strongly associated with antibody response were (in order): anosmia (OR: 14.5, 95% CI: 10.1–20.8), ageusia (OR: 10.6, 95% CI: 7.5–14), fever (OR: 3.7, 95% CI: 2.8–4.8), dyspnea (OR: 3.3, 95% CI: 2.4–4.6), all of them with a *p* < 0.0001 (Table 3).

The mean IgG level registered among seropositive patients was 43.4 AU/mL (STD 36, IQR 22–51).

**Figure 3:**
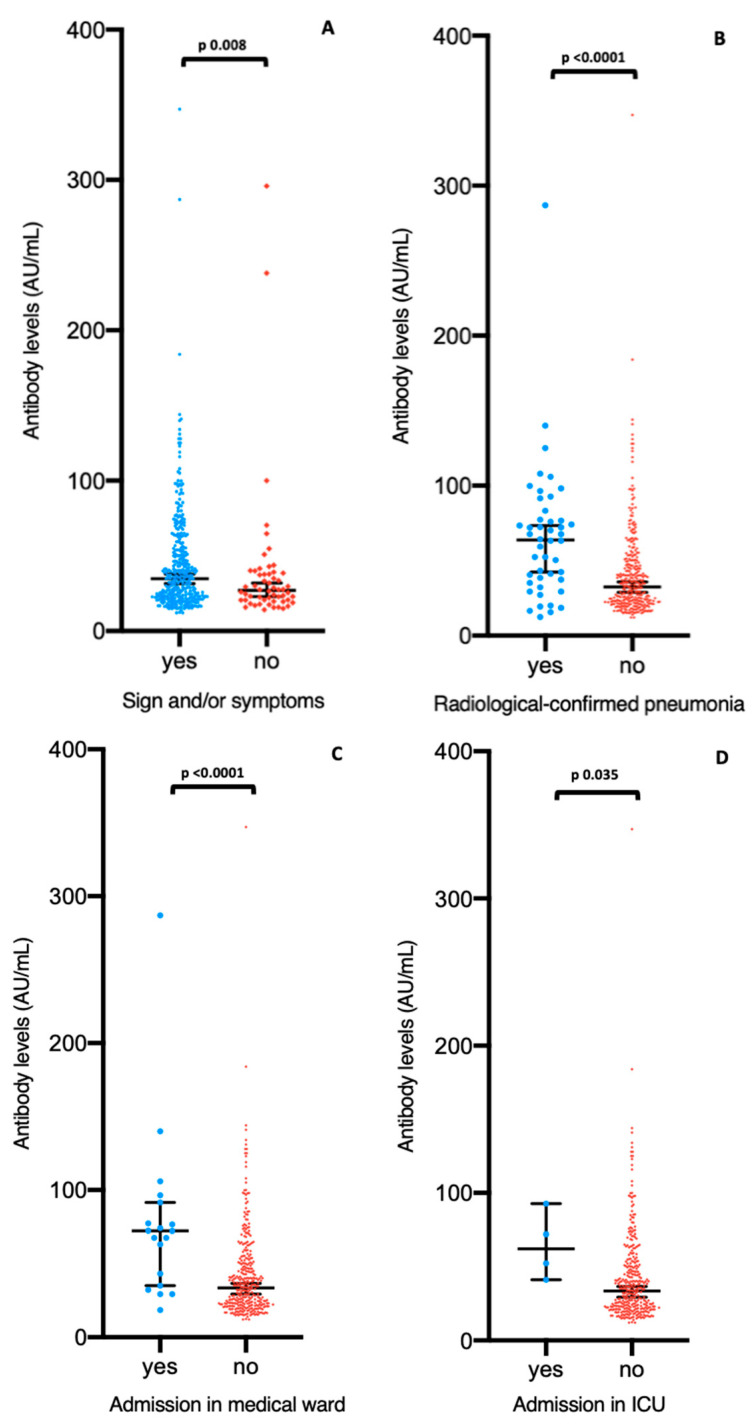
Levels (AU/mL) of IgG by symptoms (**A**), radiologic confirmed pneumonia (**B**), admission at non intensive medical ward (**C**), and admission in ICU (**D**). For (**A**), data are shown only for seropositive subjects for IgG (*n* = 433). For (**B**–**D**), data are shown only for seropositive and symptomatic subjects for IgG (*n* = 377). The center line of boxes depicts the median of IgG concentration; the lower and upper hinges correspond to 95%CI. Wilcoxon rank test was used to assess statistically significant differences in antibody levels between groups in (**A**–**D**).

IgG levels positively are significantly higher in presence of COVID-19-compatible symptoms (*p* = 0.008), radiological-confirmed pneumonia (*p* < 0.0001) and admission in medical ward (*p* < 0.0001) or in ICU (*p* = 0.035) (Figure 3).

Age (rho = 0.23 *p* value (Spearman’s correlation) < 0.0001) and signs and symptoms duration (rho = 0.11 *p* value (Spearman’s correlation) 0.028) also show a positive correlation with IgG levels.

By contrast, no association was observed between the IgG concentration and the delay time between signs and symptoms onset and the date of serology test (rho = −0.04 *p* value (Spearman’s correlation) 0.4).

The mean delay time between signs and symptoms onset and blood collection to serology test was comparable among seronegative and seropositive participants: 49 days (STD 18) in the former and 48 days (STD 14) in the latter group.

### 3.3. Cumulative Prevalence of SARS-CoV-2 Infection

Adding the 42 seronegative subjects with positive SARS-CoV-2 RT-PCR to the 433 seropositive HCWs, 475 (25.1%) participants developed COVID-19 in the study period.

## 4. Discussion

In this study we found that, as expected, having had any COVID-19 related sign or symptom is the most important factor associated with the development of SARS-CoV-2 IgG (OR: 11.3). Moreover, the likelihood of being seropositive correlates with the duration and the severity of such signs or symptoms (measured by the presence of radiologic confirmed pneumonia, the need of oxygen supplementation and the hospitalization).

Neither professional categories nor being directly involved in COVID-19 care were found to be associated with an increased risk of being seropositive probably because of the higher perception of risk among frontline HCWs [25].

As already reported [6,22], a higher IgG level correlates significantly with the presence of COVID-19-compatible symptoms, radiological-confirmed pneumonia, signs, and symptoms duration and admission in medical ward or in ICU.

Interestingly, no association was observed between the IgG concentration and the delay time between signs and symptoms onset and the date of serology test. Further studies about the dynamics of SARS-CoV-2 antibodies long after the infection could probably explain this data.

In this study, we found that around 23% of the HCWs included in the study developed SARS-CoV-2 spike protein-specific antibodies with potential neutralizing effect. This prevalence rises to 25% taking into account also those workers with SARS-CoV-2 RT-PCR confirmed infection who did not develop specific IgG response.

The seroprevalence observed is higher in comparison with the majority of similar studies recently published in Europe, Asia and US. Indeed, only Grant et al. found a higher rate (31.6%) in London UK [20] whereas all the other studies report a lower percentage of seropositivity: 0.7–9.3% [11,15,17,18,19,21,22,26,27].

The high seroprevalence recorded could be justified as follows. First of all, the Spedali Civili General Hospital has dedicated about 800 hospital beds to COVID-19 patient care situated in ICU, infectious diseases, pneumology, cardiology, neurology, nephrology, obstetric, and internal medicine units in order to better manage each patient based on the clinical picture presented. Although the PPE availability and the staff education, it is possible that the quick increase of cases in March 2020, the shortage of hospital beds and the employment of personnel inexperienced on respiratory and transmittable infections could have hindered the transmission control in the hospital wards.

Second, in a high prevalence province it is difficult to differentiate in-hospital transmission from a household transmission that, in such case, could account for a non-negligible portion of the seroprevalence registered [15]. Data from the Ministry of Health show that at the beginning of August 2020, the community seroprevalence in Lombardy was 7.5%, the highest in Italy (all the other regions was <4%) and some of the main towns of this region reached 20–25% of community seroprevalence [28]. Similarly, a seroprevalence of 10–28% were reported by seroprevalence studies among blood donors [29,30].

On the other hand, the seroprevalence observed is far away from the estimated herd immunity [31] also if measured in one of the higher prevalence settings.

One of the secondary objectives of our study was to determine the rate of asymptomatic infection among the clinical staff.

Similarly to Steensels et al. [15], we found that 87% of HCWs went through COVID-19 (positive serology) with at least one sign or symptom. Conversely, higher prevalence of asymptomatic infection has been reported by the majority of studies [18,19,26].

In early March 2020, the association between hypogeusia and hyposmia and SARS-CoV-2 infection had not been clarified. Considering as symptomatic only HCWs who suffer from the most suggestive and distinct symptoms and signs (fever, ageusia, anosmia, dyspnea, myalgia, and cough) the rate of asymptomatic HCWs among the seropositive ones increase, from 13% to 19.6%.

Due to the huge amount of job and the related stress to which our personnel was exposed during the study period, a symptomatic status could have been overreported when considering very aspecific signs and symptoms (such as fatigue, headache, gastrointestinal discomfort), therefore reducing the asymptomatic infection rate we found.

Forty-two (14.%) out of 290 HCWs previously diagnosed with SARS-CoV-2 infection did not show a detectable antibody response. We cannot discard that some HCWs may be non-responders, as found by several reports [20,22].

In conclusion, we cannot exclude a mixed effect of either serological test performance and interindividual variability of antibodies production. However, a focused analysis with a second serology check would clarify the real figure of the observed discrepancy.

This study has several limitations. Its retrospective design, coupled with the use of voluntary questionnaires asking for details about past events and the low rate of participation to the survey (around 50%), may have biased the accuracy of the information collected. Moreover, the possible presence of moral injury or post-traumatic stress syndrome occurred in HCWs [32] may have influenced the non-response rate to the questionnaire. On the other hand, we cannot exclude that the seropositive personnel could be more interested in answering to the survey and then, that this potential bias could have produced a rise in comparison to the true seroprevalence (22.9% versus 17.3%). Another important aspect is that, while several studies demonstrate that the incorrect use of PPE correlate with a higher risk of infection (and consequently with positive serology), we did not investigate PPEs use among the participants [18,19]. Third, the comparison of our data with other studies is difficult because of the different serological assay used around the laboratories.

Despite these limitations the present study has also strengths as this is one of the first studies on SARS-CoV-2 seroprevalence that involved a high number of HCWs, it was carried out in one of the geographic areas more affected in Europe at the beginning of the pandemic and it is reflected by the high seroprevalence registered.

The use of SARS-CoV-2 IgG alone (without IgM) allows to maximize specificity rather than sensitivity as suggested by CDC Guidelines for COVID-19 Antibody Testing [33]. Moreover, a serological assay able to detect the neutralizing antibodies is not only useful for epidemiologic aims but it provides a measure of the population immunity to SARS-CoV-2 [24].

Despite the available data on immunological response against SARS-CoV-2, many issues, as antibodies persistence and their protective role against reinfection, remain to be clarified.

Further studies are urgently needed to evaluate the persistence of neutralizing IgG after COVID-19. Obviously, these topics take on a great relevance for health care system preparedness in the hypothesis of long-term circulation of SARS-CoV-2.

Finally, the analysis of IgG concentration and its association with clinical and demographic variables represent an added value. Our analysis confirms that the IgG titer correlates with severity of symptoms; by contrast, we cannot exclude that asymptomatic HCWs may be low/non responders [7].

In conclusion, most participants with confirmed COVID-19 related symptoms develop an antibody response but, waiting for stronger evidence on the correlation of SARS-CoV-2 antibody production and protection against viral reinfection, seropositive HCWs should continue to correctly respect the protection measures. Furthermore, we are repeating SARS-CoV-2 IgG after six months from the first test, in order to better understand the dynamic of long-term immunity after first infection.

This study also highlights the need of regular screening by SARS CoV-2 RT-PCR for all HCWs, regardless of symptoms, to reduce the risk of hospital-acquired SARS-CoV-2 infections and its spreading and to monitor the maintenance of protective antibodies titers.

## Figures and Tables

**Table 1:** Characteristics of study participants (*n* = 1893).

Characteristic	
**Age, mean (std)**	44 (10.7)
**Female, *n* (%)**	1459 (77.1)
**Professional category, *n* (%)**	
Physicians	411 (21.7)
Nurses ^a^	818 (43.2)
Support staff	272 (14.3)
Lab technician/Other technicians	24 (1.3)
Psychologist. healthcare assistant	20 (1.1)
Other health personnel ^b^	216 (11.4)
Pharmacists. employees	132 (7)
**Working in a COVID-19 unit/ER, *n* (%)**	1008 (53.2)
**Comorbidities ^c^, *n* (%)**	613 (32.4)
**Pregnancy, *n* (%)**	22 (1.2)
**Underlying immunosuppression, *n* (%)**	45 (2.4)
**Close contact with confirmed COVID-19 case, *n* (%)**	1098 (58)
**Close contact with suspected COVID-19 case, *n* (%)**	736 (38.9)
**Reporting COVID-19 compatible symptoms, *n* (%)**	922 (48.7)
**Previously diagnosed with positive SARS-CoV-2 rt-PCR, *n* (%)**	290 (22.4) ^d^
**Hospitalization among symptomatic subject, *n* (%)**	24/922 (2.6)
Medical ward	20/922 (2.2)
ICU	4/922 (0.4)
**Radiologic evidence of viral pneumonia, *n* (%)**	48/112 ^e^ (42.9)
**Oxygen therapy, *n* (%)**	28/922 (3)

^a^ included obstetricians. scrub nurse. ^b^ included speech and physical therapist. biologists and microbiologists. ^c^ comorbidities include: obesity, cancer, diabetes, heart and liver disease, hypertension, chronic respiratory and renal disease, neurologic impairment, hematologic diseases, and other autoimmune/immunological disorders. ^d^ 290 out of 1294 test performed. ^e^ confirmed pneumonia among X-ray or CT scan performed.

**Table 2:** Distribution and strength of association as odds ratios (OR) with 95% confidence interval (95% CI) of various variables with detectable SARS-CoV-2 IgG antibodies.

Variables	Serological Test		OR (95% CI)	*p* Value
	Positive Ab (*n* = 433)	Negative Ab (*n* = 1460)		
Age, mean (std)	45 (11)	44 (11)		**0.013** ^c^
Female, *n* (%)	330 (76.2)	1129 (77.3)	0,9 (0.7;1.2)	0.6 ^d^
Working in a COVID-19 unit/ER, *n* (%)	234 (54)	774 (53)	1.04 (0.8; 1.3)	0.7 ^d^
Comorbidities, *n* (%)	141 (32.6)	472 (32.3)	1 (0.8; 1.3)	0.9 ^d^
Pregnancy, *n* (%)	7 (1.6)	15 (1)	1.6 (0.6; 3.9)	0.3 ^d^
Underlying immunosuppression, *n* (%)	12 (2.8)	33 (2.3)	1.2 (0.6; 2.4)	0.6 ^d^
Chronic steroid therapy, *n* (%)	4 (0.9)	18 (1.2)	0.7 (0.2; 2.2)	0.8 ^d^
Other immunosuppressive therapy, *n* (%)	7 (1.6)	14 (1)	1.7 (0.7; 4.2)	0.3 ^d^
COVID-19 compatible signs/symptoms, *n* (%)	377 (87)	545 (37.3)	11.3 (8.4; 15.2)	**<0.0001** ^d^
Duration of signs and symptoms (in days), mean (std)	25 (24)	20 (21)		**0.001** ^c^
Delay time between signs and symptoms onset and serology test (in days), mean (std)	48 (14)	49 (18)		0.3 ^c^
Previously diagnosed with positive SARS-CoV-2 RT-PCR ^a^, *n* (%)	248 (61)	42 (4.7)	10.1 (6.7; 15.2)	**<0.0001** ^d^
Radiologic evidence of viral pneumonia ^b^, *n* (%)	46 (12.2)	2 (0.37)	37.7 (9; 156.4)	**<0.0001** ^d^
Oxygen therapy ^b^, *n* (%)	17 (4.5)	1 (0.2)	25 (3.4; 193)	**<0.0001** ^d^
Hydroxychloroquine treatment, *n* (%)	63 (14.5)	23 (1.6)	10.6 (6.5; 17.4)	**<0.0001** ^d^
Close contact with confirmed COVID-19 case, *n* (%)	284 (65.6)	814 (55.7)	1.5 (1.2; 1.9)	**<0.0001** ^d^
Close contact with suspected COVID-19 case, *n* (%)	231 (53.4)	505 (34.6)	2.2 (1.7; 2.7)	**<0.0001** ^d^

^a^ 1294 SARS-CoV-2 rt-PCR performed: 887 IgG negative, 407 IgG positive ^b^ among the 922 symptomatic subjects (545 IgG negative, 377 IgG positive). ^c^ Mann–Whitney test. ^d^ Chi-squared test.

**Table 3:** Distribution and strength of association as odds ratios (OR) with 95% confidence interval (95% CI) of signs and symptoms with detectable SARS-CoV-2 IgG antibodies.

	Serological Test		OR (95% CI)	*p*-Value
	Negative Ab	Positive Ab		
**COVID-19 compatible symptoms**	**545**	**377**	**11.3 (8.4; 15.2)**	**<0.0001**
Fever, *n* (%)	167 (30.6)	234 (62)	3.7 (2.8; 4.8)	**<0.0001**
Fatigue, *n* (%)	341 (62.6)	314 (83.3)	3 (2.2; 4.1)	**<0.0001**
Headache, *n* (%)	278 (51)	214 (56.8)	1.3 (0.9; 1.6)	0.09
Myalgia, *n* (%)	215 (39.4)	245 (65)	2.9 (2.2; 3.7)	**<0.0001**
Cough, *n* (%)	175 (31.1)	183 (48.5)	2 (1.5; 2.6)	**<0.0001**
Dyspnea, *n* (%)	70 (12.8)	124 (32.8)	3.3 (2.4; 4.6)	**<0.0001**
Anosmia, *n* (%)	47 (8.6)	218 (57.8)	14.5 (10.1; 20.8)	**<0.0001**
Ageusia, *n* (%)	59 (10.8)	212 (56.2)	10.6 (7.5; 14.8)	**<0.0001**
Conjunctivitis, *n* (%)	73 (13.4)	42 (11.1)	0.8 (0.5; 1.2)	0.4
Rhinitis, *n* (%)	162 (29.7)	117 (31)	1.1 (0.8; 1.4)	0.7
Gastrointestinal discomfort, *n* (%)	157 (28.8)	149 (39.5)	1.6 (1.2; 2.1)	**0.001**
Dermatological lesions, *n* (%)	18 (3.3)	30 (8)	2.5 (1.4; 4.6)	**0.002**

## Data Availability

Not applicable.

## References

[1] Zhu N., Zhang D., Wang W., Li X., Yang B., Song J., Zhao X., Huang B., Shi W., Lu R. (2020). A novel coronavirus from patients with pneumonia in China, 2019. N. Engl. J. Med..

[2] Guan W., Ni Z., Hu Y., Liang W., Ou C., He J., Liu L., Shan H., Lei C., Hui D.S.C. (2020). Clinical characteristics of coronavirus disease 2019 in China. N. Engl. J. Med..

[3] Tabata S., Imai K., Kawano S., Ikeda M., Kodama T., Miyoshi K., Obinata H., Mimura S., Kodera T., Kitagaki M. (2020). Clinical characteristics of COVID-19 in 104 people with SARS-CoV-2 infection on the Diamond Princess cruise ship: A retrospective analysis. Lancet Infect. Dis..

[4] Oran D.P., Topol E.J. (2020). Prevalence of Asymptomatic SARS-CoV-2 Infection. Ann. Intern. Med..

[5] Wei W.E., Li Z., Chiew C.J., Yong S.E., Toh M.P., Lee V.J. (2020). Presymptomatic Transmission of SARS-CoV-2-Singapore. Morb. Mortal Wkly Rep..

[6] Long Q.-X., Liu B.-Z., Deng H.-J., Wu G.-C., Deng K., Chen Y.-K., Liao P., Qiu J.-F., Lin Y., Cai X.-F. (2020). Antibody responses to SARS-CoV-2 in patients with COVID-19. Nat. Med..

[7] Xiang F., Wang X., He X., Peng Z., Yang B., Zhang J., Zhou Q., Ye H., Ma Y., Li H. (2020). Antibody Detection and Dynamic Characteristics in Patients with Coronavirus Disease 2019. Clin. Infect. Dis..

[8] Dati della Sorveglianza Integrata COVID-19 in Italia. https://www.epicentro.iss.it/coronavirus/sars-cov-2-dashboard.

[9] Piva S., Filippini M., Turla F., Cattaneo S., Margola A., De Fulviis S., Nardiello I., Beretta A., Ferrari L., Trotta R. (2020). Clinical presentation and initial management critically ill patients with severe acute respiratory syndrome coronavirus 2 (SARS-CoV-2) infection in Brescia, Italy. J. Crit. Care.

[10] Rizzi M., Castelli F., Latronico N., Focá E. (2020). SARS-CoV-2 invades the West. How to face a COVID-19 epidemic in Lombardy, Northern Italy?. Infez. Med..

[11] Iversen K., Bundgaard H., Hasselbalch R.B., Kristensen J.H., Nielsen P.B., Pries-Heje M., Knudsen A.D., Christensen C.E., Fogh K., Norsk J.B. (2020). Risk of COVID-19 in health-care workers in Denmark: An observational cohort study. Lancet Infect. Dis..

[12] World Health Organization Transmission of SARS-CoV-2: Implications for Infection Prevention Precautions. https://www.who.int/publications/i/item/modes-of-transmission-of-virus-causing-covid-19-implications-for-ipc-precaution-recommendations.

[13] Centers for Disease Control and Prevention Interim Infection Prevention and Control Recommendations for Healthcare Personnel During the Coronavirus Disease 2019 (COVID-19) Pandemic. https://www.cdc.gov/coronavirus/2019-nCoV/hcp/infection-control.html.

[14] Black J.R.M., Bailey C., Przewrocka J., Dijkstra K.K., Swanton C. (2020). COVID-19: The case for health-care worker screening to prevent hospital transmission. Lancet.

[15] Steensels D., Oris E., Coninx L., Nuyens D., Delforge M.-L., Vermeersch P., Heylen L. (2020). Hospital-Wide SARS-CoV-2 Antibody Screening in 3056 Staff in a Tertiary Center in Belgium. JAMA.

[16] Poulikakos D., Sinha S., Kalra P.A. (2020). SARS-CoV-2 antibody screening in healthcare workers in a tertiary centre in North West England. J. Clin. Virol..

[17] Fusco F.M., Pisaturo M., Iodice V., Bellopede R., Tambaro O., Parrella G., Di Flumeri G., Viglietti R., Pisapia R., Carleo M.A. (2020). COVID-19 among healthcare workers in a specialist infectious diseases setting in Naples, Southern Italy: Results of a cross-sectional surveillance study. J. Hosp. Infect..

[18] Stubblefield W.B., Talbot H.K., Feldstein L.R., Tenforde M.W., Rasheed M.A.U., Mills L., Lester S.N., Freeman B., Thornburg N.J., Jones I.D. (2020). Seroprevalence of SARS-CoV-2 Among Frontline Healthcare Personnel during the First Month of Caring for Patients with COVID-19—Nashville, Tennessee. Clin. Infect. Dis..

[19] Self W.H., Tenforde M.W., Stubblefield W.B., Feldstein L.R., Steingrub J.S., Shapiro N.I., Ginde A.S., Prekker M.E., Brown S.M., Peltan I.D. (2020). Seroprevalence of SARS-CoV-2 Among Frontline Health Care Personnel in a Multistate Hospital Network—13 Academic Medical Centers, April–June 2020. Morb. Mortal. Wkly. Rep..

[20] Grant J.J., Wilmore S.M.S., McCann N.S., Donnelly O., Lai R.W.L., Kinsella M.J., Rochford H.L., Patel T. (2020). Seroprevalence of SARS-CoV-2 antibodies in healthcare workers at a London NHS Trust. Infect. Control Hosp. Epidemiol..

[21] Lahner E., Dilaghi E., Prestigiacomo C., Alessio G., Marcellini L., Simmaco M., Santino I., Orsi G.B., Anibaldi P., Marcolongo A. (2020). Prevalence of Sars-Cov-2 infection in health workers (HWs) and diagnostic test performance: The experience of a teaching hospital in central Italy. Int. J. Environ. Res. Public Health.

[22] Garcia-Basteiro A.L., Moncunill G., Tortajada M., Vidal M., Guinovart C., Jiménez A., Santano R., Sanz S., Méndez S., Llupià A. (2020). Seroprevalence of antibodies against SARS-CoV-2 among health care workers in a large Spanish reference hospital. Nat. Commun..

[23] World Health Organization (2020). Global Surveillance for COVID-19 Caused by Human Infection with COVID-19 Virus: Interim Guidance. https://apps.who.int/iris/handle/10665/331506.

[24] Bonelli F., Sarasini A., Zierold C., Calleri M., Bonetti A., Vismara C., Blocki F., Pallavicini L., Chinali A., Campisi D. (2020). Clinical and analytical performance of an automated serological test that identifies S1/S2-neutralizing igG in COVID-19 patients semiquantitatively. J. Clin. Microbiol..

[25] Peres D., Monteiro J., Almeida M.A., Ladeira R. (2020). Risk perception of COVID-19 among Portuguese healthcare professionals and the general population. J. Hosp. Infect..

[26] Schmidt S.B., Grüter L., Boltzmann M., Rollnik J.D. (2020). Prevalence of serum IgG antibodies against SARS-CoV-2 among clinic staff. PLoS ONE.

[27] Korth J., Wilde B., Dolff S., Anastasiou O.E., Krawczyk A., Jahn M., Cordes S., Ross B., Esser S., Lindemann M. (2020). SARS-CoV-2-specific antibody detection in healthcare workers in Germany with direct contact to COVID-19 patients. J. Clin. Virol..

[28] ISTAT, Salute M della (2020). Primi Risultati dell’indagine di Sieroprevalenza sul SARS-CoV-2. https://www.istat.it/it/files//2020/08/ReportPrimiRisultatiIndagineSiero.pdf.

[29] Percivalle E., Cambiè G., Cassaniti I., Nepita E.V., Maserati R., Ferrari A., Di Martino R., Isernia P., Mojoli F., Bruno R. (2020). Prevalence of SARS-CoV-2 specific neutralising antibodies in blood donors from the Lodi Red Zone in Lombardy, Italy, as at 06 April 2020. Eurosurveillance.

[30] Valenti L., Bergna A., Pelusi S., Facciotti F., Lai A., Tarkowski M., Berzuini A., Caprioli F., Santoro L., Baselli G. (2020). SARS-CoV-2 seroprevalence trends in healthy blood donors during the COVID-19 Milan outbreak. medRxiv.

[31] Fontanet A., Cauchemez S. (2020). COVID-19 herd immunity: Where are we?. Nat. Rev. Immunol..

[32] Shaukat N., Ali D.M., Razzak J. (2020). Physical and mental health impacts of COVID-19 on healthcare workers: A scoping review. Int. J. Emerg. Med..

[33] Centers for Disease Control and Prevention (2020). Coronavirus Disease 2019 (COVID-19) Interim Guidelines for COVID-19 Antibody Testing Current Status of Antibody Testing in the United States Antigenic Targets. https://www.cdc.gov/coronavirus/2019-ncov/lab/resources/antibody-tests-guidelines.html.

